# Snakes on a slope: strong anti-gravitactic responses and differential habitat use in the Saharan horned viper (*Cerastes cerastes*) in the Negev desert

**DOI:** 10.1098/rsos.221652

**Published:** 2023-03-22

**Authors:** Arik Dorfman, Aziz Subach, Inon Scharf

**Affiliations:** School of Zoology, George S. Wise Faculty of Life Sciences, Tel Aviv University, Tel Aviv 69978, Israel

**Keywords:** gravitaxis, giving-up density, habitat selection, sand dune, slope, tracking

## Abstract

The way species use their habitat dictates their intra- and interspecific interactions. We studied the effects of the microhabitat type and slope on the movement behaviour of the Saharan horned viper (*Cerastes cerastes*) in its natural habitat. This viper occurs in sand dunes and moves mostly by sidewinding. Additionally, we studied the microhabitat preference of desert rodents—the vipers' main prey. We placed the vipers on different natural dune slopes and recorded their behaviour. We found a strong anti-gravitactic response: vipers moved more frequently towards the top of the dune than in any other direction, despite a decrease in stride length with increasing slope. The foraging-related behaviour of the vipers was concentrated in the dune semi-stable areas rather than its stable or shifting sand areas. We measured rodent activity by placing seed trays in the dune allowing the rodents to collect seeds. Rodent activity was the highest in the shifting sands, closely followed by the semi-stable microhabitat. These results suggest the vipers use the semi-stable microhabitat mainly for foraging and may use the shifting sand areas as commuting routes between such areas. This study may be of use for conservation efforts of psammophilic species in desert dunes.

## 1. Introduction

An animal's habitat can be defined as a set of environmental factors vital for a species’ survival and reproduction [1]. If species that co-occur in the same habitat are too similar in their niche, one may drive the other to extinction. However, when species differ in their habitat use, such as by preferring distinct microhabitats or being active during different parts of the day, coexistence is more likely (spatial or temporal partitioning; [2,3]). Furthermore, habitats are rarely homogeneous and different microhabitats within a habitat possess distinct environmental features. Such microhabitats can be used by the same species for different purposes. For example, within a marine bird's oceanic habitat, rocky islands are used for nesting, upwellings or shelf edges are used as feeding grounds, and corridors of directional winds enable commuting between the former two [4,5].

Habitat use depends on many factors. Food availability, for example, and its interplay with other biotic factors, like predation, parasite load and competition, define the habitat profitability for foraging [6–9]. Movement costs affect habitat use too, as many species use areas in the habitat that enable them to minimize their energy expenditure while travelling [10]. Coyotes (*Canis latrans*), for example, avoid areas with deep snow [11], and Atlantic puffins (*Fratercula arctica*) let tidal currents drift them to their feeding grounds instead of flying there [12]. Finally, areas used as shelters have specific features and may even be the limiting factor for the distribution range of species [13,14].

A species’ differential use of its habitat affects, in turn, the community-level interactions. For example, a predator's spatial distribution of its foraging activity creates a ‘landscape of fear’ that affects the habitat use of its prey species [15]. Consequently, deciphering the habitat use pattern is the first step in the study of interspecific interactions. We focused here on the habitat use of the Saharan horned viper (*Cerastes cerastes*) in a dune habitat and its predator–prey interactions with dune-dwelling rodents.

Dunes are rather simple habitats, they are sparsely vegetated, and animal tracks are easily imprinted upon their sandy surface. All of which facilitate field observations and experiments [7,16]. Plant cover and the dune's slope are the two main traits of the dune microhabitats, and both may affect the habitat use of dune-dwelling animals. Dunes comprise three main microhabitats separable according to their plant cover and stabilization [17]: (i) *The stable areas*, usually at the bottom of the dune, are relatively densely vegetated and the soil is covered with biogenic crust (a thin layer of soil hardened by fungus, moss, etc.). (ii) *The semi-stable areas*, usually at the dune slopes, are more sparsely vegetated, and the crust covers a smaller proportion of the soil and there are ripples in the sand caused by the wind. (iii) *Shifting sands*, usually at the slopes and the dune top, are sparsely vegetated with almost no annuals or crust; the sand there is the most mobile and ripples cover most of the area.

Regarding the dune's slope, dunes are sand hills with maximal slopes of approximately 34° [18]. Inevitably, dune-dwelling animals spend much of their paths going uphill or downhill [19]. The greater the mass of the animal, the more energy it spends moving uphill [20]. Thus, the dune's slope may set a challenge to larger animals but be irrelevant or even advantageous for smaller ones [21]. The effect of slope on animal movement and behaviour was studied in several vertebrate groups [10,20,22–24]. For example, both field and laboratory studies have shown that animals avoid moving diagonally to the slope [19,25]. This may facilitate three-dimensional navigation by receiving balanced vestibular cues [25] or help to avoid unbalanced muscular activity [19]. Additionally, the species' natural habitat plays a role in their reaction to the slope: species that live naturally in a complex environment are better at vertical exploration than those that live on plains [26]. Some species are also inclined to go down or up the slope—gravitaxis or anti-gravitaxis, respectively [24,27,28].

The biomechanics of a convergent species of our study species (*C. cerastes*), the sidewinder (*Crotalus cerastes*), has been thoroughly studied on various sand slopes in the laboratory [29,30]. However, no field study has been conducted on the effect of slope on the behaviour of any dune-dwelling snake, to our knowledge. Our research question was whether the natural behaviour of *C. cerastes* is affected by the slope in its natural habitat. We examined this question by placing vipers on different slopes in the dune's open area and recorded their reactions. Hence, the viper had a choice to move in random directions (regardless of the slope) or move preferably to a specific direction (upwards, downwards or sidewards). Aside from the vipers’ direction of movement, their velocity and stride length were also recorded to test whether the slope influences either of them. We expected the slope to slow down the vipers if they decide to move upwards, but it should help them move faster downwards. The fact that this study was conducted in the field ensured that the conditions in our experiment are within the range that the vipers naturally encounter. Thus, the results of the experiment are more relevant to the vipers' natural behaviour in their habitat, than those of a sterile, laboratory experiment.

**Figure 1.**
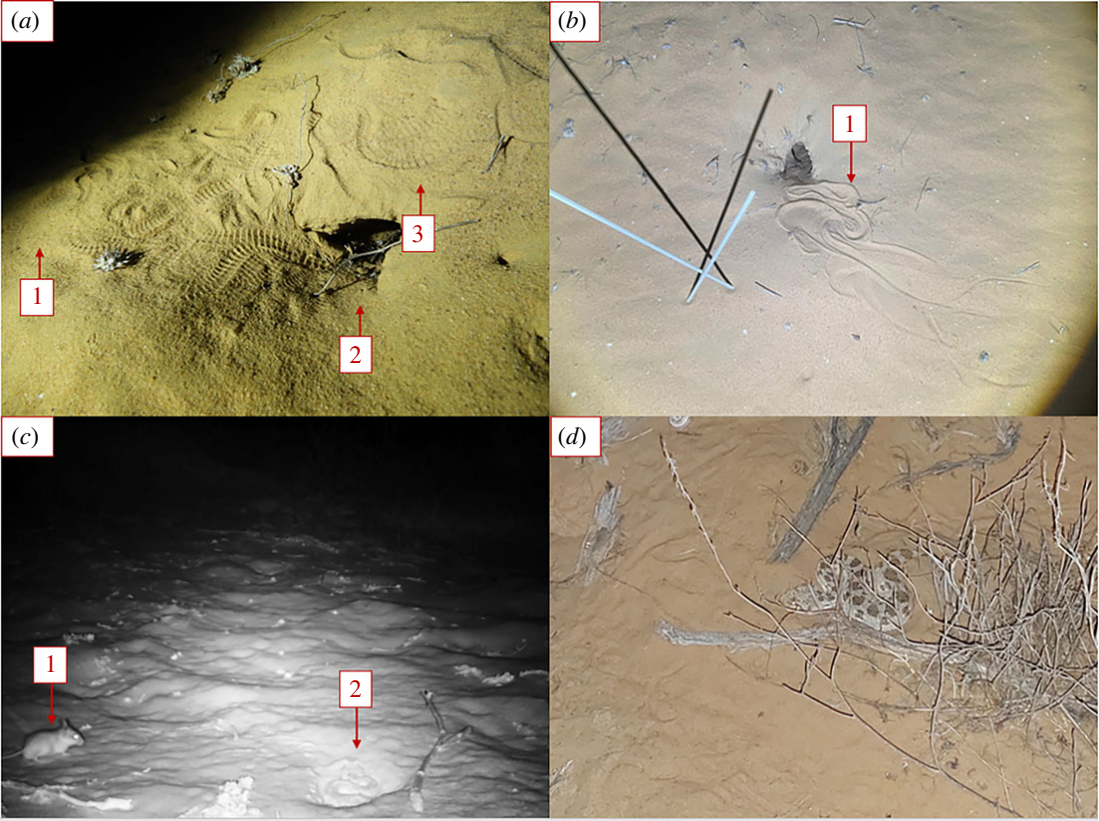
Tracks of foraging-related activities (*a*,*b*), and vipers engaging in these activities (*c*,*d*). These findings were used in the analysis of the viper habitat selection. (*a*) Burrow scan: (1) tracks leading to a rodent's burrow, (2) tracks entering the burrow, (3) tracks leaving the burrow. (*b*) Tracks of a viper in an ambush position: (1) the ambush tracks were usually found at the entrance of a rodent's burrow. (*c*) A snapshot of a trail camera showing a viper ambushing in a burrow's entrance: (1) a gerbil and (2) a viper. (*d*) A viper in an ambush position under a bush.

In a previous study, we found that the vipers possess no microhabitat preference for any of the three dune microhabitats described above [31]. However, foraging-related activities were not considered. Signs of foraging can be easily distinguished by interpreting the vipers’ tracks in the sand. The tracks of the distinct, coiled ambush position and tracks going in and out of a burrow can be accurately identified (figure 1). The age of the tracks can be determined as strong afternoon winds obscure most tracks from the previous night [32]. In this study, we accounted for these activities and re-examined the microhabitat preference of the viper for foraging activities.

In parallel, we tested for the microhabitat preferences of the rodents in the dune while foraging. Rodents are plausibly the main prey of such and similar *Cerastes* vipers [33], and the rodents' spatial activity thus indicates where *C. cerastes* should focus its foraging effort*.* In other words, determining their habitat preferences may shed light on the vipers’ habitat use. The rodent community in the Negev sand dunes has been studied extensively, particularly the two most abundant species, *Gerbillus pyramidum* and *Gerbillus andersoni* [34]. Both primarily prefer the semi-stable microhabitat while foraging and they coexist through temporal and habitat partitioning [2,35]. However, the studies mentioned above were performed in an adjacent area to our study site, where the dunes were more stabilized, homogeneous, and the rodents' predation risk might be different. As foraging decisions are highly flexible and depend on factors like predation risk [36,37], we aimed to verify these findings and tested which microhabitat (the stable, semi-stable or shifting sands) the rodents preferred, specifically in our study site.

## 2. Materials and methods

### 2.1. Study site

The study was conducted in a roughly 2 × 2 km habitat, which comprised two linear dunes along the east–west axis in the sand dunes of the northwestern Negev, Israel (Holot Agur; 30.9280° N, 34.4130° E) (electronic supplementary material, figure S1). The area is the easternmost extension of the Sahara Desert. The annual rainfall in 2022 was 59.3 mm but the mean annual rainfall is somewhat higher (68.9 ± 35.0 mm; Israel Meteorological Service; https://ims.gov.il/en). Maximum and minimum temperatures in August were 41.0° and 18.6°, respectively, and in January 24.1° and −0.2°, respectively (Meteo-tech; http://www.meteo-tech.co.il/negev/negev_daily.asp). After the first rains in December, annual plants emerge, and most plants wither by May. Among the annual species in the study site are *Erucaria pinnata, Cutandia memphitica, Matthiola livida, Neurada procumbens, Plantago cylindrica, Cyperus conglomeratus* and *Atractylis carduus.* Among the perennial bushes are *Stipagrostis scoparia, Anabasis articulata, Heliotropium digynum, Moltkiopsis ciliata, Thymelea hirsute* and *Retama raetam* (personal observations).

**Figure 2.**
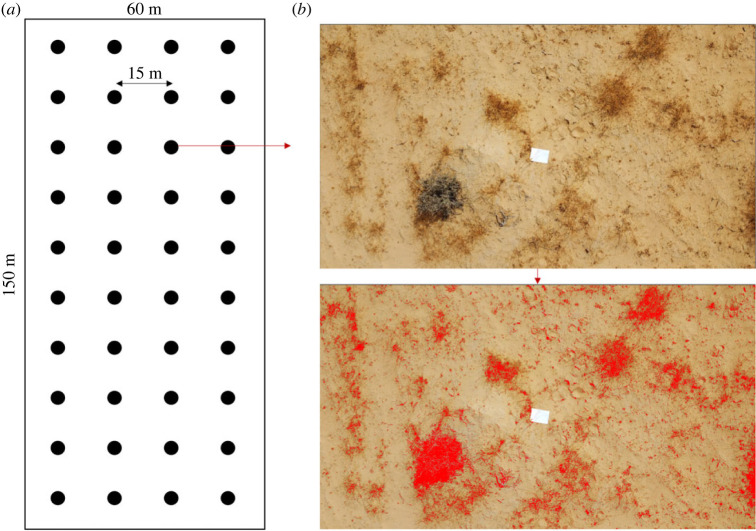
(*a*) A scheme of a grid comprising 40 stations in a regular spatial pattern, with 15 m between adjacent ones. (*b*) An example of the plant cover analysis (station 1.3.3): in each drone photo, we calculated the plant cover by calculating the proportion of pixels in plant-covered areas out of the whole photo. The red arrow indicates a magnification of a single station.

**Figure 3.**
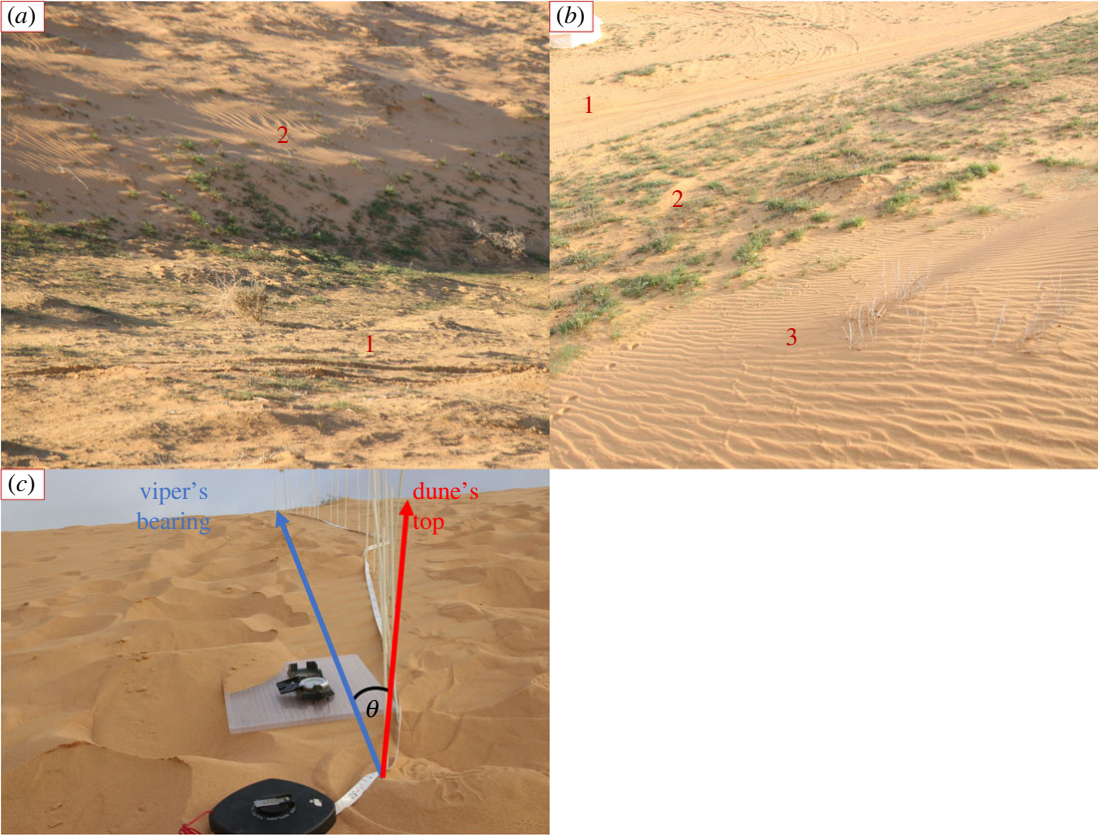
(*a*,*b*): The three microhabitats in the dune, stable (1), semi-stable (2) and shifting sands (3), (*a*) from the dune's bottom: the ripples in the semi-stable are clearly shown, and (*b*) from the dune's top: all the microhabitats are shown. (*c*) Measuring the outcome of the slope experiment: fibreglass poles and measuring tape were used to measure the distance between sidewinding strides. A compass was used to measure the movement direction of the viper (blue arrow) and the direction to the dune's top (red arrow). The angle between them (here, *θ*) was used in the angle analysis. A level mounted on a 2 m stick was used to measure the slope of the dune (not shown here).

We marked four grids (150 × 60 m) on the site. Each grid comprised 40 stations (15 m between two adjacent ones) arranged in four columns and 10 rows, while each column transecting the dune from north to south (figure 2; electronic supplementary material, figure S2). Two grids were placed on the Northern dune and two on the Southern one. Each station's coordinates were taken for later use. We measured three parameters in each station—the slope, microhabitat and plant cover. The slope was measured by a digital level (‘Kapro 393 Digi-pro’; accuracy: 0.2°) mounted on a 2 m long stick, laid on the sand along the steepest incline of the dune. The microhabitat was categorized into stable areas, semi-stable areas and shifting sands. A stable area was distinguished by the presence of annuals, developed biological crust and the absence of ripples caused by the wind; a semi-stable area by annuals and ripples; and the shifting sand by ripples and the absence of annuals (figure 3*a,b*). The plant cover was measured in April 2022 to verify that there are indeed statistical differences between the microhabitats, as stated in the literature [17]. Each station was photographed by a DJI Mavic mini drone from a 6 m height and each photo was analysed for the proportion of plant cover by the software ImageJ [38]. The plant cover at the three microhabitats was compared using one-way ANOVA and Tukey test as a *post hoc* test. The data were arcsine square-root-transformed as common for proportions [39], in order to ‘stretch out’ proportions that are close to the distribution's boundaries (0 and 1).

### 2.2. Study animal

*Cerastes cerastes* lives throughout the Sahara Desert from Morocco in the west, to south Israel and the western part of the Arabic peninsula in the east [40]. It is a relatively small viper, measuring 51.5 ± 6.6 cm snout-to-tail and weighing 107 ± 39 g (mean ± s.d.; [31]). It primarily moves by sidewinding (electronic supplementary material, video S1) and hunts either actively or by ambushing its prey. While ambushing, it usually burrows itself halfway in the sand and waits in a coiled position or lies in a coiled position inside a rodent's burrow, facing the entrance. In an active hunt, it enters and exits rodents' burrows in search for prey [31]. Both hunting modes were also described for its smaller sister taxon *Cerastes vipera*, which, however, mostly visits lizards’ rather than rodents' burrows [41].

In this study, vipers were tracked down by their unique sidewinding tracks in the sand (electronic supplementary material, figure S3). Each viper was caught, weighed, marked by UV non-toxic paint, and photographed alongside a scale for length measurement. The length was measured digitally in ImageJ (as in [31]; for a detailed discussion on digital measuring of snakes see [42]). The photographs were also used to individually recognize the vipers if their paint mark was shed along with their skin. This was possible due to the vipers’ unique pattern of spots on their body (electronic supplementary material, figure S4). At the end of the study, 19 different snakes were captured on 34 occasions. The mean weight and snout-to-tail length of the vipers tracked in this study were 120 ± 56 g and 50.98 ± 9.45 cm, respectively. One snake was captured after it had shed its skin this year (2022) and three snakes, captured in a previous study in 2021, were recaptured this year.

### 2.3. Slope experiment

In order to check for the slope effect on the vipers' behaviour, vipers were placed on different slopes on the dune and their behaviour was recorded. Twenty trials (15 different individuals) were made between late May and early September 2022. Seven females and eight males, measuring 54.12 ± 4.97 cm and weighing 132.00 ± 45.83 g were used. The trials took place on the shifting sands microhabitat, for two reasons: first, most of this microhabitat is located on the dune slopes, unlike the stable microhabitat which is mostly flat. Thus, in our study site, travelling through the shifting sands is equivalent to travelling on a slope. Second, it is the only microhabitat where the slope is even, unlike in the stable and semi-stable microhabitats where surface irregularities, caused by plants, crust and burrows, may complicate its behaviour and our interpretation of the results.

The captured vipers were transported via a bucket to shifting sand areas of the dune with varying slopes. The whole process took 10 min at most. On arrival, the bucket was turned upside-down, and the viper was left for an acclimatization period of 15 min. In the meantime, we set up night-vision recording goggles (Bresser Digital NV Binocular 3,5x w. recording Monochrome) on a tripod, downwind and as far as possible from the viper (about 6 m away). After 15 min, we started recording, lifted the bucket from the viper and walked back to the binoculars. The vipers were tracked as long as possible (5.6 ± 3.8 min; mean ± s.d.) without moving the tripod, in order not to disturb them. Then, the vipers were recaptured, and their tracks were marked by placing poles in the sand on top of the head imprint of each track. The path length and the length between a sample of consecutive tracks (stride length) were measured by a measuring tape. We documented the movement direction of the viper and the direction to the dune's top by a compass and calculated the angle between the two. The angle from the dune's top was defined by the angle between the line connecting the start and end of the viper's path to the line connecting the start of the viper's path and the top of the dune (figure 3*c*). The path's slope was measured by a digital level mounted on a 2 m long stick. The videos were used to determine the vipers’ velocity. Most vipers remained immobile at the beginning of the trials, scanned the area by tongue-flicking, oriented themselves to their desired direction and only then began to crawl in a sidewinding gait. The travel time was, therefore, calculated by measuring the time from the viper's first sidewinding until it reached the end of the measured path. The path distance was then divided by the travel time to calculate the velocity.

Additionally, we compared the velocities in the slopes of the shifting sand to the velocities achieved in a horizontal stable microhabitat. Nine of the vipers used for the experiment were taken to dense vegetation patches in the stable microhabitat and tested under the same protocol as described above. The vipers were all using a lateral undulation gait in the stable area and were followed until they regained their usual sidewinding gait as they got out of the vegetated areas. The vipers were tested first on the shifting sands and then on the stable microhabitat, which may have tired or discouraged the vipers in the second trial. However, this was probably not the case, as we allowed them to move only a small distance at their own pace, while it takes more than a few trials to exhaust a snake that is forced to move at full speed [43–45]. The experiment was conducted in this fashion, as we prioritized to minimize the varying factors in the primary slope experiment over the stable versus shifting sands one, as the differences between treatments were much subtler in the former one and more susceptible to be obscured by background noise. All vipers were transported back to their capture site and released at the end of the experiments in no more than 1.5 h.

The viper's handling and our presence had minimal effects on its behaviour, for several reasons. *Cerastes cerastes* reacts to danger in the following manner: it freezes in place, and if the danger has not passed, it buries itself in the sand. If the intruder approaches, the viper starts rubbing its scales, making hiss-like noises, and eventually tries to crawl away or bite (personal observations). In no experiment did a viper get past the freezing stage. Additionally, the vipers were not moving at their maximal velocity during the trials. This became evident at the end of the trials when the vipers were approached to be recaptured. All vipers bolted away from us more quickly than during the trial.

To determine if the vipers had a preferred direction in relation to the dune and if the slope affected this preference, we performed the following analysis: the direction to the top of the dune was set as the 0° in each trial and the viper's movement direction was measured in relation to that direction. We calculated the mean angle to the top of the dune and its confidence intervals (*n* = 18, CI = 95%; as the angles were not measured in the two first trials due to technical issues). To test the significance of the result, we used a Rayleigh test for the uniformity of distribution, under the null hypothesis that the angles are distributed uniformly through the circle and the alternative hypothesis that the angles are clumped in a particular direction [46]. To test the effect of the slope on the angle to the top of the dune, we fitted a mixed linear model to the data. The dependent variable was the absolute value of the angles (as both negative and positive values express deviation from the dune top); the fixed variables were the mean slope in the trial, the number of hours after sunset when the trial took place, the moon phase (full or new moon), mass and the viper's sex. The identity of the viper was considered a random variable. To detect the effect the slope had on the vipers' velocity and stride length, both were fitted in a mixed-effect linear model each (*n* = 20). The fixed and random variables were the same as in the angle model. In all models, non-significant factors were excluded from analysis one by one, with the least significant factor removed, until the most significant factor remained. The velocity in the shifting sand was compared with that in the annuals using a paired *t*-test (*n* = 9). All vipers were tested only twice, once in each microhabitat.

### 2.4. Microhabitat selection

Our goal here was to examine whether *C. cerastes* prefers a specific microhabitat for foraging and daily shelter or whether its selection is random. Vipers were tracked and their paths were followed back to the nightly starting location. While following the tracks, every case where tracks of foraging-related behaviour—burrow scan or ambush—were spotted, was marked by a pole, and its coordinates were noted. These behaviours were chosen because their tracks can be easily distinguished, and they represent two foraging modes of the vipers—active hunting and sit-and-wait hunting ([31,41]; figure 1). The nightly starting locations’ coordinates were also taken. The path endpoints were only included when the viper's tracks were found after the viper had retired for the daily resting place. These were included in the analysis because vipers forage and take shelter in the same type of place—inside or near burrows, and the two behaviours are often not separable [41].

A total of 46 locations were documented. However, only a single location from a specific viper per night was randomly chosen to be considered in the analysis, reducing the number to 20 locations from 11 different individuals. Consequent nights, when the same viper was sampled, had at least a two-week interval between them, thus we regarded these data points as independent measurements. The microhabitat surrounding each of the locations of these foraging-related behaviours was classified as stable/semi-stable/shifting sand, as described in the ‘*Habitat characterization’* section. These data were used to answer the question of which microhabitat the vipers prefer by categorically classifying the microhabitats in the viper-chosen locations. Additionally, part of the locations was photographed by a drone (from 6 m in height) on the following morning, to verify our categorical classifications by measuring the plant cover in the same locations. These images were analysed for their proportion of plant cover by ImageJ. For comparison, random coordinates (*n* = 21) were generated using Python. These coordinates were then visited in the field and underwent the same treatment as the viper-chosen locations.

The microhabitat choice was compared using two complementary tests: for the categorical microhabitat classification, a *χ*^2^ test was used to compare the viper-chosen locations (*n* = 20) to an expected distribution derived from the randomly generated locations (*n* = 21). We repeated this analysis 10 times, each time choosing a single location from a list of locations visited by an individual viper on a specific night, randomly. This was done to ensure that our results are robust, and the same result is obtained when using slightly different data. For the continuous data (the plant cover measurements that were analysed from drone photographs), a Welch two-sample *t*-test was used to compare the viper-chosen locations (*n* = 13; as not all locations were photographed) and the randomly generated locations (*n* = 20). The plant cover data were square-root-transformed as they deviated from a normal distribution.

### 2.5. Rodent activity

We used the grids mentioned in the first section (see ‘*Study site*’; figure 2) to describe the spatial distribution of the foraging activity of rodents across the three dune microhabitats. This measurement took place on three nights in late August and early September. Each night, a grid was chosen, and aluminium trays (31 × 43 × 3.5 cm) filled with 3 l of sifted sand containing 3 g seeds were placed in every grid station (*n* = 80 per night). Seed trays were used in many studies to evaluate granivores' foraging activity [6–8,47]. Two columns in each grid were filled with wheat seeds and two with millet, as seed size could potentially affect foraging decisions [48]. At dawn, the presence of rodent tracks at the trays was noted to verify foraging by rodents and not by other granivores (like ants or birds). The remaining seeds were sifted and weighed to the nearest 0.1 mg using an electronic scale (XT 220A, Precisa Gravimetrics, Dietikon, Switzerland) in the laboratory. The weight of the remaining seeds in each tray can be referred to as the rodents’ giving-up density (GUD). GUD is the amount of food left in a patch after the forager has left it, and it reflects the balance of benefits and costs/risks the patch holds [6,47]. The GUDs left in the grids were fitted in a multiple linear model. The independent variables were microhabitat (three types), seed type (wheat versus millet), slope (continuous) and plant cover (continuous). Non-significant factors were excluded from the analysis one by one until only significant factors remained.

All statistical analyses in this study were conducted in R statistical software [49] except for the circular statistics in the slope experiment, which was calculated manually.

## 3. Results

### 3.1. Habitat characterization

**Figure 4.**
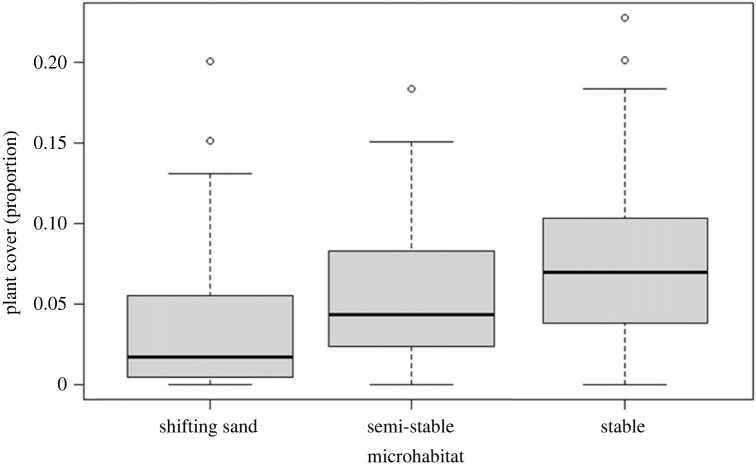
A boxplot of plant cover proportions across the different microhabitats. The midline represents the median, and the upper and lower limits of the box are the third and first quartile, respectively. The whiskers extend to 1.5 times the interquartile range from the top and bottom of the box to the furthest datum within that distance.

We chose the maximal slope in each column of each aspect in each grid to measure the mean maximal slopes that the vipers encounter in their habitat. The mean maximal slope in the dune was 15.48° ± 7.31° (mean ± 1 s.d.; *n* = 32). The mean plant cover in the dune, as measured in April 2022, was 5.90% ± 4.88% (mean ± 1 s.d.; *n* = 160). The mean (±1 s.d.) plant cover in the stable, semi-stable, and shifting sand was 7.68% ± 5.09%, 5.54% ± 4.27% and 3.83% ± 4.70%, respectively. The plant cover differed across microhabitats (ANOVA: *F*_2,157_ = 11.116, *p* < 0.001; figure 4). The stable microhabitat was significantly more vegetated than the other two and the semi-stable was significantly more vegetated than the shifting sands (Tukey test: shifting sand × stable: *p* < 0.001; shifting sand × semi-stable: *p* = 0.029; stable × semi-stable: *p* = 0.034). See the electronic supplementary material for the dataset.

### 3.2. Slope experiment

Twenty trials (15 different individuals) were made in the shifting sands in late May and early June 2022. Nine of these vipers were also examined in the stable microhabitat for the sake of comparison. The angle analysis consisted of 18 repetitions (the angle to the top of the dune was not measured in the first two trials for technical issues). All but three vipers moved straight up the dune while the remaining three moved upwards and sideways: the mean angle to the dune's top was 1.72° ± 13.23° (mean ± 95% CI). Rayleigh's test rejected the null hypothesis of a uniform angle distribution with high certainty (*z*_18_ = 14.753, *p* < 0.001). The only significant factor in the mixed linear model was the moon phase (mixed linear model: moon: *t* = −4.366, *p* = 0.016; hours after sunset: *t* = −5.728, *p* = 0.101; sex: *t* = −0.485, *p* = 0.637; mass: *t* = −0.080, *p* = 0.938; slope: *t* = −0.044, *p* = 0.966): vipers crawled more straight up when there was a full moon. The effect size, however, was small, and the difference between the means (Cohen's D) was 0.48 s.d.

**Figure 5.**
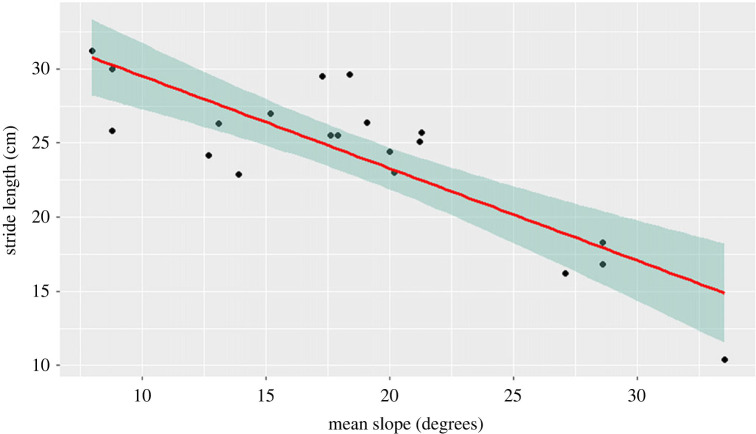
Results of the mixed linear models fitted on the stride length of vipers on the shifting sands. The red line represents the regression line and the green area represents the confidence intervals (95%) of the regression line. As the dune's slope increases (*x*-axis), the stride length decreases (*y*-axis). The graph was plotted using the R package ggplot2 (v. 3.3.5; [50]).

Two other mixed linear models were fitted to the experiment in the shifting sands, where the stride length and velocity were set as the response variable, one each time. The non-significant variables in all models were gradually excluded from the analysis. The stride length was log-transformed to achieve a normal distribution of the model's residuals. The slope significantly decreased the stride length (mixed linear model: slope: *t* = −13.980, *p* < 0.001; hours after sunset: *t* = 0.295, *p* = 0.800; sex: *t* = −1.402, *p* = 0.184; mass: *t* = 1.646, *p* = 0.131; moon: *t* = −1.131, *p* = 0.302; figure 5) but had no effect on the velocity (mixed linear model: hours after sunset: *t* = 0.426, *p* = 0.677; sex: *t* = 0.735, *p* = 0.481; mass: *t* = −0.079, *p* = 0.938; moon: *t* = 0.450, *p* = 0.677; slope: *t* = −1.715, *p* = 0.104) as well as all other variables examined for stride length.

The mean stride length in the shifting sand was 24.19 ± 5.20 cm (mean ± 1 s.d.). The mean velocity in the shifting sands and the mean velocity in the stable microhabitat were 5.47 ± 1.65 cm s^−1^ and 0.69 ± 0.40 cm s^−1^, respectively. The velocity in the shifting sands was significantly higher than that in the stable microhabitat (t_9_ = −7.86, *p* < 0.001). As seen from the mean velocities, the vipers were almost an order of magnitude faster in the shifting sands.

### 3.3. Microhabitat selection

**Figure 6.**
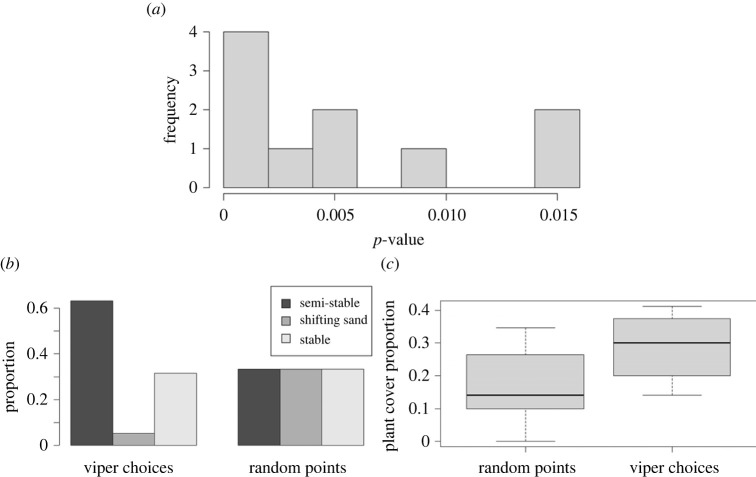
Results of microhabitat selection analysis. (*a*) A histogram of 10 resamples of the habitat selection analysis: each time, uncorrelated viper-chosen locations were randomly selected and tested via *χ*^2^ test to fit an expected distribution derived from the microhabitat in the randomly generated locations. The histogram plots the *p*-values of these tests. The *p*-values are on the *x*-axis and frequency is on the *y*-axis. All 10 *χ*^2^ tests came significant. (*b*) A bar plot of one of the samples compared with the randomly generated locations. In this sample, 12 locations were recorded on the semi-stable, one on the shifting sands and six on the stable. (*c*) Plant cover proportions in locations selected by the vipers, compared with random points. The plant cover was higher in viper-chosen locations compared with random locations.

Twenty locations from 11 different vipers were considered in the analysis so that each viper was sampled only once each night to avoid correlated samples. These locations were randomly resampled 10 times to ensure the analysis was robust. We sampled in the field 21 random points generated by a computer for comparison. Of these, seven were found in the semi-stable area, seven in the stable area and seven in the shifting sands. Thirteen viper-chosen locations and 20 random points were photographed by a drone for plant cover analysis. Out of 10 resamples, the foraging-related activity was significantly higher in the semi-stable area than in the other microhabitats, based on a *χ*^2^ test (figure 6*a*,*b*). The plant cover in the viper-chosen locations was higher than expected by random (*t*_23.393_ = −3.134, *p* = 0.005; figure 6*c*)

### 3.4. Rodent activity

**Figure 7.**
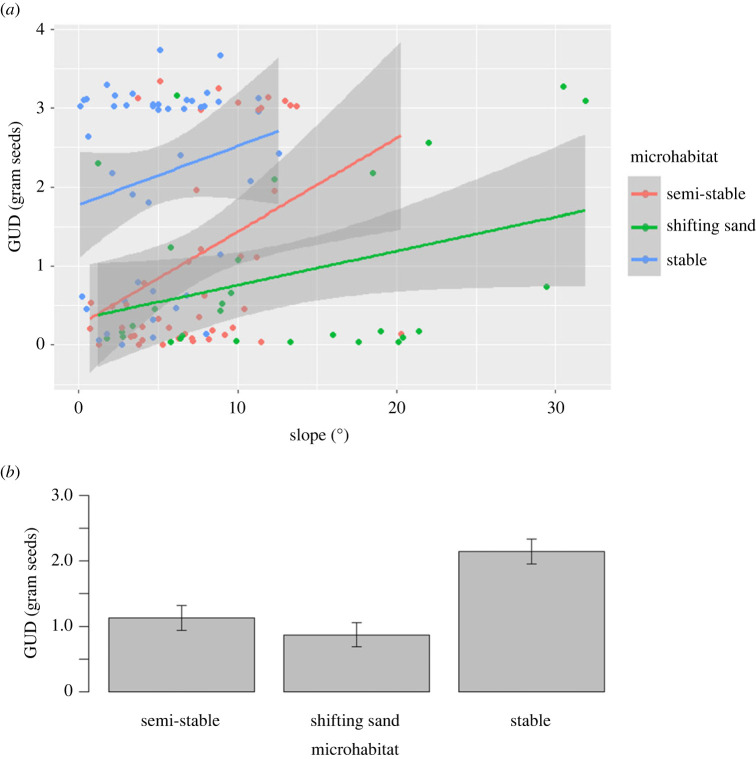
Results of the rodent activity analysis. (*a*) The effect of slope and microhabitat on the GUD in gram seeds. The effect of slope is only significant when considering the different microhabitats. The blue, red and green lines represent the regression line in the stable, semi-stable and shifting sands microhabitats, recpectively. The grey areas represent the confidence interval (95%) of the regression lines. The graph was plotted using the R package ggplot2 (v. 3.3.5; [50]). (*b*) The effect of microhabitat on GUD. All microhabitats are significantly different from each other (see text for *p*-values). Most seeds were taken from the shifting sands, followed by the semi-stable microhabitat, and the higher GUD was recorded on the stable microhabitat. The error bars represent ± 1 s.d. from the mean.

Seed type and plant cover did not affect the GUD and were therefore excluded from the analysis (*t* = 0.236, *p* = 0.814; *t* = 0.713, *p* = 0.477; respectively). The slope had a positive effect on the GUD (*t* = 3.174, *p* = 0.002; figure 7*a*). Microhabitats differed from each other (shifting sands × semi-stable: *t* = −1.968, *p* = 0.049; stable × semi-stable: *t* = 4.67, *p* < 0.001; stable × shifting sands: *t* = 5.528, *p* < 0.001). Most seeds were taken in the shifting sands, closely followed by the semi-stable microhabitat (figure 7*b*).

## 4. Discussion

The tested dune-dwelling viper *C. cerastes* demonstrated strong anti-gravitactic responses: in 20 trials no viper moved downhill and all but three moved nearly straight up. This is despite a decrease in stride length with the increasing slope. The velocity in the inclined shifting sands, however, was greater than the velocity in the flat stable area by nearly an order of magnitude. The foraging-related activity of the vipers was concentrated in the semi-stable area. These results suggest a differential use of the habitat for *C. cerastes*, which resembles that of its rodent prey. While the vipers use the semi-stable areas for foraging, the shifting sand area may be used as convenient commuting routes between patches.

Vipers were going straight up the dune, and not diagonally to the slope. Going straight up or down as a response to an increasing slope was demonstrated in rodents and lizards [19,25]. Both groups avoid moving diagonally on a tilted surface. Also, avoiding going down but not up, on steep, sandy slopes was shown for the desert lizard *Uma scoparia* [19]. Maybe, in steep slippery slopes, like those found on a sand dune, the risk of slipping into unknown terrain is perceived as riskier than moving up. A detailed examination of the videos elaborates on the experiment's results: most vipers had their head oriented toward the bottom of the dune at the start of the trial and repositioned themselves to face upwards before they started to move. This detail suggests that the vipers considered all available directions before deciding to move uphill.

Despite the anti-gravitactic response, we found a negative effect of the slope on the stride length (figure 5), but with no effect on velocity. The result matches a biomechanical laboratory study with another sidewinder (*Crotalus cerastes*) [29]. In these experiments, the snakes compensate for the slippage caused by the increasing slope by increasing their contact with the ground, resulting in shorter strides but not a decrease in velocity. The fact that the slope forced the vipers to advance in smaller steps did not affect the vipers' choice of moving up. Perhaps, the effort of making more steps with increasing slope is not considered high for the vipers, but a metabolic study involving sidewinding movement on different slopes is required to confirm this suggestion. It is also worth noting that the main effect was due to trials done on extreme slopes (29°–37°) (figure 5), while the mean maximal slope in our study site is more moderate 15.48° ± 7.31°. This suggests that in average conditions, the vipers are not affected energetically by their habitat's slope.

Vipers moved slightly more upwards on moonlit nights than on dark nights. Moonlight indeed affects the activity level and patterns of *C. cerastes*, as they lack pit-organs and probably rely more heavily on the sense of sight than convergent sidewinders [51]. However, as the effect size in our experiment is small (the difference between the mean angle to the dune's top on full-moon nights versus no-moon nights is only 0.48 s.d.), more detailed research is needed to assess the effect of moonlight on the vipers’ anti-gravitaxis.

Vipers were nearly an order of magnitude faster on shifting sand than on the densely covered stable area—a result that confirms our former experiment where viper velocity inside an experimental patch of ‘annual plants’ was compared with that on the shifting sands [31]. This is despite the different measuring methods used in both studies and the fact that in the former one, vipers moved on a flat, not inclined patch of shifting sands. We always tested vipers on the stable area after they were tested on the shifting sands, which might have tired or discouraged the vipers in the second trial. However, an effect size as large as we observed between the treatments (Cohen's D = −3.977) probably cannot be attributed to the vipers' fatigue alone. Additionally, as the vipers were approached to be collected after the second trial, they had enough stamina to bolt away from the experimenter (personal observations).

Most of the foraging activity and shelter sites of vipers were in the semi-stable microhabitat (figure 6). Indeed, ambushing snakes usually choose a favourable place for ambushing [52]. The semi-stable area is rich in rodent activity relative to the stable areas (figure 7*b*), supplies cover (plants and burrows), which is much scarcer in the shifting sands, may camouflage the viper from its own predators and its prey, and still provides many spots containing loose sand where vipers can bury themselves in ambush. As noted, the rodents’ activity was concentrated in the shifting sands and semi-stable microhabitats of the dune (figure 7*b*). The lowest GUD was observed in the shifting sands and not in the semi-stable microhabitat, as was stated in the literature [2,35], although the difference between these two microhabitats was minor. This may be a result of a high predatory risk from snakes, which hunt near cover, and a low predatory risk from owls, which hunt better in the open [37]. Owls were never spotted in the study site during the research.

These results, together with the fact that the stable areas lie at the bottom of the dune [17], suggest that vipers may use the shifting sand area as a convenient commuting route and the semi-stable area as their main ‘hunting grounds’. Other sidewinding snakes avoid densely vegetated areas as they travel [53], and sidewinding is speculated to be an adaptation for moving on loose substrates, like sand or mud [54]. The most densely vegetated areas in the dune are the stable areas at the dune's bottom. Thus, slope and lack of vegetation are bounded environmental factors in this habitat. Anti-gravitaxis in *C. cerastes* may represent an adaptation for moving towards more open areas, where these sidewinding snakes are morphologically adapted to move efficiently, and from where they can more easily reach distant patches of semi-stable areas—where they hunt. Another possibility is that vipers moved upwards to better orient themselves in the new environment they were transferred to [55]. Whatever the interpretation of the viper's goals might be, the fact that they were inclined to go up the dune indicates that the slope has a positive effect on the vipers' movement decisions.

The stable area appears to be the least favourable microhabitat for *C. cerastes*, even though in our previous study [31] we found no difference in the habitat selection of the vipers across the three microhabitats. This contradiction can be settled by considering a yet unstudied trait of the vipers’ behaviour—stealth. In our experiments, vipers were forcibly exposed in an open area. Most of them chose to go up the dune, maybe as the easier path to a distant patch of vegetation away from the site (an important clarification is that the vipers were probably not reacting to an immediate danger but to an exposure, similar to that of an ambush failure; [56]). However, when a viper starts its activity in a vegetated area it would probably benefit from staying under cover until it finds a suitable ambush site. Thus, by moving into the stable microhabitat vipers remain unexposed, but at an increased movement cost. This hypothesis should be studied experimentally, as it may emphasize the different use and importance of all available dune microhabitats to this predator.

## 5. Conclusion

Dunes in Israel are an endangered environment. Fast-growing settlements threaten the last resort of many dune specialists [57]. Our study emphasizes the fact that predators in the dunes, and probably other animals as well, use the dune microhabitats differently. Thus, to support these species, the whole array of microhabitats should be conserved. The study of habitat use by predators and prey can improve our understanding of their interactions. We found that although vipers move faster on the shifting sand area their main foraging efforts concentrate on the semi-stable areas. Thus, the semi-stable areas should be the riskiest microhabitat for rodents. Additionally, there are more predators of rodents in the dunes that may compete with *C. cerastes*, such as red foxes (*Vulpes vulpes*) and diadem snakes (*Spalerosophis diadema*) [58,59]. Comparing their habitat use with that of *C. cerastes* may reveal mechanisms of coexistence, similar to those revealed for other species in the habitat [35,60].

## Data Availability

The dataset is attached as an Excel file. The data are provided in the electronic supplementary material [61].
